# Supplementary material for: A molecularly defined basalo-prefrontal-thalamic circuit regulates sensory and affective dimensions of pain in male mice
Source: Nat Commun. 2026 Jan 29;17:2134. doi: 10.1038/s41467-026-69001-2 (PMC12957525; doi:10.1038/s41467-026-69001-2)
Supplement: Supplementary file 2 — Reporting Summary [file 41467_2026_69001_MOESM2_ESM.pdf]

nature portfolio

Corresponding author(s): Yi Zhang

Last updated by author(s): Dec 23, 2025

Reporting Summary

Nature Portfolio wishes to improve the reproducibility of the work that we publish. This form provides structure for consistency and transparency in reporting. For further information on Nature Portfolio policies, see our Editorial Policies and the Editorial Policy Checklist.

Statistics

For all statistical analyses, confirm that the following items are present in the figure legend, table legend, main text, or Methods section.

n/a	Confirmed	
☐	☒	The exact sample size (*n*) for each experimental group/condition, given as a discrete number and unit of measurement
☐	☒	A statement on whether measurements were taken from distinct samples or whether the same sample was measured repeatedly
☐	☒	The statistical test(s) used AND whether they are one- or two-sided<br>*Only common tests should be described solely by name; describe more complex techniques in the Methods section.*
☒	☐	A description of all covariates tested
☒	☐	A description of any assumptions or corrections, such as tests of normality and adjustment for multiple comparisons
☐	☒	A full description of the statistical parameters including central tendency (e.g. means) or other basic estimates (e.g. regression coefficient) AND variation (e.g. standard deviation) or associated estimates of uncertainty (e.g. confidence intervals)
☐	☒	For null hypothesis testing, the test statistic (e.g. *F*, *t*, *r*) with confidence intervals, effect sizes, degrees of freedom and *P* value noted<br>*Give P values as exact values whenever suitable.*
☒	☐	For Bayesian analysis, information on the choice of priors and Markov chain Monte Carlo settings
☒	☐	For hierarchical and complex designs, identification of the appropriate level for tests and full reporting of outcomes
☒	☐	Estimates of effect sizes (e.g. Cohen's *d*, Pearson's *r*), indicating how they were calculated

Our web collection on statistics for biologists contains articles on many of the points above.

Software and code

Policy information about availability of computer code

Data collection	We listed all softwares used in the experiments and for analysis in the Methods section.<br>In vivo miniscopic calcium imaging, the data were acquired with nVoke system (Inscopix, CA, USA).<br>For open field test and conditioned place preference test, movement was recorded using Med Associates ENV-510 (Med Associates, St. Albans, VT, USA).<br>For optogenetic stimulation, laser was generated by OEM Lasers/OptoEngine machine.<br>IHF and FISH images were collected using a Zeiss LSM800 confocal microscope or Keyence BZ-X810 microscope.
Data analysis	We listed all softwares used in the experiments and the data analysis has been described in the Methods section. Images (including those for FISH and histology) were analyzed using ImageJ (V1.54p). All miniscopic calcium imaging data were analyzed with Inscopix Data Processing Software (Version 1.9, Inscopix, CA, USA) and custom-written Matlab code. Statistical analyses were performed and plotted with Matlab or GraphPad Prism software 8.

For manuscripts utilizing custom algorithms or software that are central to the research but not yet described in published literature, software must be made available to editors and reviewers. We strongly encourage code deposition in a community repository (e.g. GitHub). See the Nature Portfolio guidelines for submitting code & software for further information.

Data

Policy information about availability of data

All manuscripts must include a data availability statement. This statement should provide the following information, where applicable:

- Accession codes, unique identifiers, or web links for publicly available datasets
- A description of any restrictions on data availability
- For clinical datasets or third party data, please ensure that the statement adheres to our policy

Behavioral annotation and statistics source data has been provided for each figure. The custom code that supports the findings from this study are available from the corresponding authors upon request.

Research involving human participants, their data, or biological material

Policy information about studies with human participants or human data. See also policy information about sex, gender (identity/presentation), and sexual orientation and race, ethnicity and racism.

Reporting on sex and gender	N/A
Reporting on race, ethnicity, or other socially relevant groupings	N/A
Population characteristics	N/A
Recruitment	N/A
Ethics oversight	N/A

Note that full information on the approval of the study protocol must also be provided in the manuscript.

Field-specific reporting

Please select the one below that is the best fit for your research. If you are not sure, read the appropriate sections before making your selection.

- [x] Life sciences
- [ ] Behavioural & social sciences
- [ ] Ecological, evolutionary & environmental sciences

For a reference copy of the document with all sections, see nature.com/documents/nr-reporting-summary-flat.pdf

Life sciences study design

All studies must disclose on these points even when the disclosure is negative.

Sample size	No statistical methods were used to pre-determine sample sizes. Our sample sizes are similar to those reported in previous publications (PMID: 38291284, 36548429, 37734381).
Data exclusions	Mice that, after histological inspection, had the location of the viral injection (reporter protein) or of the optic fiber(s) outside the area of interest, were excluded.
Replication	All behavioral experiments were independently repeated at least twice. The representative fluorescent images were chosen from two to four independent experiments. To ensure that results are reproducible, we included in the methods all details relative to softwares, reagents, and protocols. We plotted individual data points and provided source data. Information about the statistical test used is reported in the figure legends.
Randomization	All animals are housed in the same room with ambient temperature and proper light-dark cycles. Animals with similar age and body weights were chosen as experimental subjects. All mice were randomly assigned to different groups.
Blinding	All investigators were blinded to group allocation during data collection and analysis.

Reporting for specific materials, systems and methods

We require information from authors about some types of materials, experimental systems and methods used in many studies. Here, indicate whether each material, system or method listed is relevant to your study. If you are not sure if a list item applies to your research, read the appropriate section before selecting a response.

Materials & experimental systems

n/a	Involved in the study
☐	☒ Antibodies
☒	☐ Eukaryotic cell lines
☒	☐ Palaeontology and archaeology
☐	☒ Animals and other organisms
☒	☐ Clinical data
☒	☐ Dual use research of concern
☒	☐ Plants

Methods

n/a	Involved in the study
☒	☐ ChIP-seq
☒	☐ Flow cytometry
☒	☐ MRI-based neuroimaging

Antibodies

Antibodies used	Rabbit anti-cFos (1:1000, Synaptic Systems, #226003)<br>Rabbit anti-ChAT (1:1000, Milliopore, #ZRB1012-25UL)<br>Donkey anti-rabbit Alexa Fluor 488 (Thermo Scientific, #A21206)<br>Donkey anti-rabbit Alexa Fluor 568 (Thermo Scientific, # A10042)
Validation	All antibodies used in this study were obtained from commercial suppliers and were validated by the manufacturers. The validation is reported on their website.

Animals and other research organisms

Policy information about studies involving animals; ARRIVE guidelines recommended for reporting animal research, and Sex and Gender in Research

Laboratory animals	Male mice of at least 2 months of age were used in all the experiments. The wild-type C57BL/6 mice (Jax, 000664), B6.Cg-Foxp2tm1.1(cre)Rpa/J mice (Jax, 030541) and B6.129S6-Chattm2(cre)lowl/J mice (Jax, 006410) were acquired from Jackson Laboratory.
Wild animals	The study did not involve wild animals.
Reporting on sex	Behavior tests were performed using male mice.
Field-collected samples	This study did not involve field-collected samples.
Ethics oversight	All experiments were conducted in accordance with the National Institute of Health Guide for Care and Use of Laboratory Animals and approved by the Institutional Animal Care and Use Committee (IACUC) of Boston Children's Hospital and Harvard Medical School.

Note that full information on the approval of the study protocol must also be provided in the manuscript.

Plants

Seed stocks	N/A
Novel plant genotypes	N/A
Authentication	N/A
